# Quantitative evaluation of T-cell repertoire restoration following hematopoietic stem cell transplantation in patients with and without graft versus host disease

**DOI:** 10.3389/fimmu.2026.1778172

**Published:** 2026-03-03

**Authors:** Xin Zhang, Pengyi Song, Longhai Tang, Xiangjun Ji, Xiaohui Hu, Xiao Ma, Feng Chen, Xuefeng He, Jun Wang, Weiyang Li, Huiying Qiu, Shengli Xue, Ying Wang, Miao Miao, Suning Chen, Depei Wu, Xiaowen Tang, Mingyuan Wang, Wenbo Wang, Xiaopeng Tian

**Affiliations:** 1 National Clinical Research Center for Hematologic Diseases, The First Affiliated Hospital of Soochow University, Suzhou, China; 2 Institute of Blood and Marrow Transplantation, Collaborative Innovation Center of Hematology, Soochow University, Suzhou, China; 3 Department of Hematology, The First Affiliated Hospital of Soochow University, Jiangsu Institute of Hematology, Suzhou, China; 4 Department of Chemical and Biological Engineering, Northwestern University, Evanston, IL, United States; 5 Research Laboratory, Suzhou Blood Center, Suzhou, China; 6 Department of Oncology, Shanghai Tenth People’s Hospital, Tongji University School of Medicine, Shanghai, China; 7 AI Center for Immunosequencing, LeaLing Biopharma Co., Ltd., Suzhou, China

**Keywords:** hematopoietic stem cell transplantation, graft versus host disease, next-generation sequencing, T cell receptors, T cell reconstitution

## Abstract

**Introduction:**

Acute graft-versus-host disease (aGVHD) is a major cause of mortality following hematopoietic stem cell transplantation (HSCT). Its pathogenesis is primarily driven by alloimmune T cells, which recognize host tissues as foreign. A deeper characterization of the T cell receptor (TCR) repertoires in patients with aGVHD could provide critical insights into the disease's mechanisms and identify potential predictive biomarkers.

**Methods:**

We employed next-generation sequencing to comprehensively profile the T cell receptor alpha (TRA) and beta (TRB) chains in HSCT recipients, comparing those with and without aGVHD. Our analysis focused on defining the functional kinetics of TCR clones, monitoring changes in overall T-cell diversity through the identification of complementarity-determining region 3 (CDR3) sequences, and quantifying the extent of clonal expansion within the T-cell population.

**Results:**

Our analysis revealed that TCR repertoires exhibited significantly increased diversity in patients with active aGVHD compared to those without. Notably, this elevated diversity was dynamic and decreased as the symptoms of aGVHD improved clinically. Furthermore, TCR clustering analysis identified the presence of common TCR repertoire signatures among different patients who developed aGVHD, suggesting shared antigen-driven T cell responses.

**Discussion:**

The dynamic changes and shared signatures within the TCR repertoire are closely associated with the development and resolution of aGVHD. These findings indicate that monitoring the TCR repertoire could serve as a valuable tool for predicting the onset of aGVHD. This approach holds promise for facilitating more personalized diagnostic and treatment strategies for aGVHD, and potentially for other T-cell-mediated pathologies.

## Background

Allogeneic hematopoietic stem cell transplantation (allo-HSCT) represents a vital therapeutic strategy for numerous hematological and immunological conditions (1). T cell reconstitution following HSCT has been a central topic in immunology. In the initial post-transplant period, donor T cells proliferate via a thymus-independent pathway could potentially induce graft-versus-leukemia effects and trigger acute graft-versus-host disease (aGVHD). The latter is a significant cause of morbidity and mortality, thereby constraining the broader application of HSCT (2, 3).

T-cell reconstitution is essential for determining clinical outcomes. However, the complexity of the T-cell repertoire makes it difficult to predict the occurrence of GVHD in clinical practice (4). The comprises millions of T cell clones, each bearing unique T cell receptors (TCRs) composed of distinct α and β subunits. These unique TCR subunits are generated by the recombination of gene segments on the α and β TCR loci, termed TRA and TRB, located on chromosomes 14q and 7q, respectively (5, 6). Despite decades of research, the identity and activity of the alloreactive donor T cells that cause aGVHD have remained inaccessible to clinical practitioners, largely due to the highly personalized and complex repertoire of TCR hypervariable regions that arise when unique T cell donors are transplanted into recipients with unique histocompatibility antigens. Over the past 2 decades, several methods have been implemented for the characterization of the T cell recognition spectrum. Early investigations evaluating spectra classification strategies consistently demonstrated the long-lasting skewing of post–allo–HSCT TCR repertoires (7, 8). The development of next-generation sequencing has facilitated the comprehensive classification of the recombined TCR complementarity-determining region 3 (CDR3) repertoire sequences and enabled the quantitative analyses of patient-specific TCR and facilitated its use post-HSCT (9–11).

TCR is a pivotal molecule on the surface of T cells, crucial for antigen recognition and T cell activation. The diversity and specificity of the TCR render may facilitate its use as a diagnostic biomarker for GVHD. However, further research is required to confirm its potential (12). The assessment of the TCR composition and alterations post-transplant could be used to predict the onset and progression of GVHD. Therefore, in this study, we used repertoire sequencing to identify the dominant serum T-cell clones in patients with or without aGVHD at 21 to 100 days post-allo-HSCT, and in healthy individuals (HIs). In addition, we also determined the differences in the TCR diversity between the patients with and without aGVHD and the similarities in the TCR repertoires in aGVHD patients. The findings of this analysis were used to identify the potential of TCR repertoires that could be used to predict aGVHD.

## Results

### Landscape of TCR repertoires in patients with or without GVHD

For this study, we separately harvested approximately 8 ml of blood samples from 15 patients who developed GVHD and 13 patients who did not develop GVHD (no GVHD) at 21 to 100 days following allo-HSCT. In addition, we also collected samples from 15 HIs. The study design and patient characteristics are described in more detail in **Table 1**. The immune repertoire information of these patients is illustrated in **Supplementary Table 1**.

**Table 1:** Patients' characteristics.

Patients	Age/sex	Disease^a^	Disease status at HSCT^b^	Donor age / sex	Type of donor^c^	Conditioning Regimen^d^	GVHD prophylaxis^e^	GVHD/ grade
GVHD								
Patient 01	49/M	MDS	PR	M/22	Haplo	MAC	MMF-CSA-ATG-MTX	III-IV
Patient 02	18/M	ALL	CR	F/43	Haplo	MAC	CSA-MMF-MTX	I-II
Patient 03	57/M	CMML	PR	M/31	Haplo	MAC	CSA-ATG-MTX	I-II
Patient 04	45/F	CML-AP	CR	F/54	MSD	MAC	CSA-ATG-MTX	I-II
Patient 05	56/M	MDS	PR	F/60	MSD	MAC	CSA-ATG-MTX	I-II
Patient 06	46/F	AML	CR	M/25	Haplo	MAC	MMF-CSA-ATG-MTX	I-II
Patient 07	20/M	ALL	CR	M/49	Haplo	MAC	MMF-CSA-ATG-MTX	III-IV
Patient 08	45/F	AML	CR	F/19	Haplo	MAC	CSA-ATG-MTX	I-II
Patient 09	42/F	AML	PR	F/47	MSD	MAC	CSA-MTX	III-IV
Patient 10	58/F	AML	PR	M/31	Haplo	MAC	CSA-MMF-ATG-MTX	I-II
Patient 11	55/M	AML	CR	M/33	Haplo	MAC	CSA-ATG-MTX	I-II
Patient 12	58/M	AML	CR	M/23	MUD	MAC	CSA-ATG-MTX	III-IV
Patient 13	60/M	AML	PR	F/34	Haplo	MAC	MMF-CSA-ATG-MTX	III-IV
Patient 14	32/M	SAA	PR	M/60	Haplo	MAC	MMF-CsA-MTX	I-II
Patient 15	49/M	PMF	PR	M/22	Haplo	MAC	MMF-CSA-ATG-MTX	III
No GVHD								
Patient 18	29/F	AML	CR	M/53	Haplo	MAC	CSA-ATG-MTX	
Patient 19	55/F	MDS	PR	M/30	Haplo	MAC	MMF-CSA-ATG-MTX	
Patient 20	37/M	PMF	PR	M/10	Haplo	MAC	MMF-CSA-ATG-MTX	
Patient 21	19/M	AML	CR	F/22	MSD	MAC	MMF-CSA-MTX	
Patient 22	59/F	AML	CR	M/40	MUD	MAC	MMF-CSA-ATG-MTX	
Patient 23	48/F	ALL	CR	M/25	Haplo	MAC	CSA-ATG-MTX	
Patient 24	27/M	CAA	PR	M/34	Haplo	MAC	CSA-ATG-MTX	
Patient 25	38/M	AML	CR	M/15	Haplo	MAC	CsA-MMF-MTX	
Patient 26	46/M	AML	CR	F/42	MSD	MAC	CSA-MTX	
Patient 27	32/M	AML	PR	M/53	Haplo	MAC	MMF-CSA-ATG-MTX	
Patient 28	50/M	AML	CR	M/25	Haplo	MAC	CSA-ATG-MTX	
Patient 29	30/M	AML	CR	F/30	Haplo	MAC	MMF-CSA-ATG-MTX	
Patient 30	27/M	AML	CR	M/24	MUD	MAC	MMF-CSA-ATG-MTX	
GVHD-pre								
Patient 31	17/M	AML	CR	M/40	MMUD	MAC	MMF-CSA-ATG-MTX	III-IV
Patient 32	20/M	T-LBL	PR	M/48	Haplo	MAC	CSA-ATG-MTX	III-IV
Patient 33	42/F	AML	PR	M/14	Haplo	MAC	MMF-CSA-ATG-MTX	I-II

^a^MDS, Myelodysplastic syndrome; ALL, Acute lymphoblastic leukemia; CMML, Chronic myelomonocytic leukemia; CML-AP, Chronic myeloid leukemia– accelerated phase; AML, Acute myeloid leukemia; S/CAA, Serious/ Chronic aplastic anemia, PMF, Primary myelofibrosis: T-LBL, T lymphoblastic leukemia/lymphoma. ^b^PR, Partial response; CR, Complete response. ^c^MSD, Matched sibling donor; MUD, Matched unrelated donor, MMUD, Mismatched unrelated donor; ^d^MAC, Myeloablative conditioning. ^e^MMF, Mycophenolate mofetil; CSA, Cyclosporine-A, ATG, Anti-human thymocyte globulin, MTX, Methotrexate.

**Figure 1:**
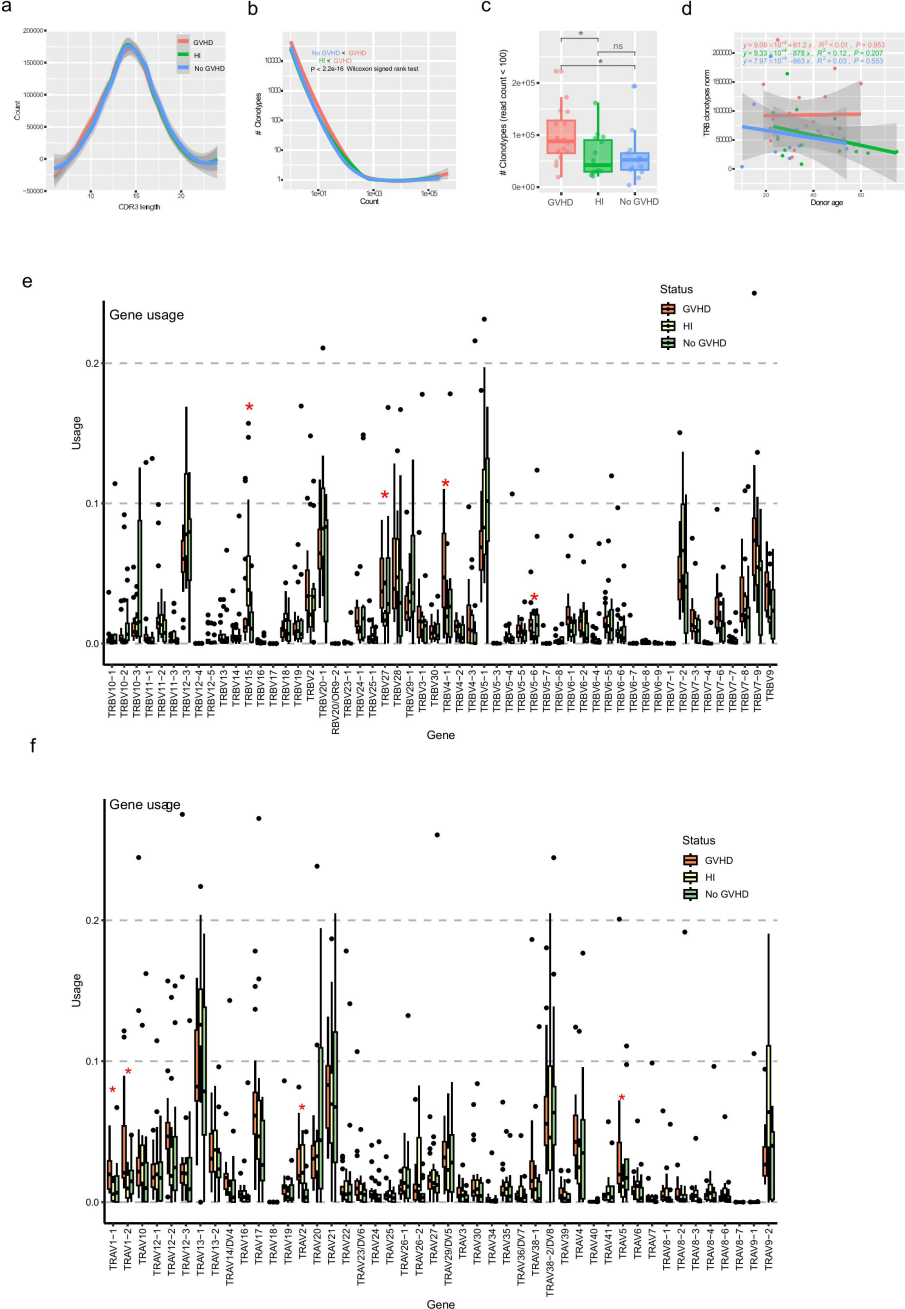
Overview of peripheral blood TRB repertoire features in patients with or without aGVHD and healthy individuals. **(a)** Distribution of TRB CDR3 amino acid lengths across all productive reads in each group, addressing whether gross structural features of the repertoire differ between GVHD, no-GVHD, and healthy individuals. Shaded areas indicate 95% confidence intervals from LOESS smoothing. **(b)** Clonotype abundance distributions of the TRB repertoire after normalization, visualizing the overall structure of clonal frequency spectra and the relative contribution of low-frequency clonotypes in each group (Kolmogorov–Smirnov test and Wilcoxon signed rank test). This panel provides a descriptive comparison of repertoire architecture rather than a quantitative diversity metric. **(c)** Number of low-frequency clonotypes (read count < 100) per sample in each group. The numbers of low-frequency clonotypes in patients with GVHD were significantly higher than those observed in patients without GVHD. *p<0.05; ns, not significant. **(d)** Relationship between donor age and TRB clonotype richness in patients and healthy individuals, examining whether age-associated effects contribute to observed repertoire differences. **(e, f)** Relative usage of TRB **(e)** and TRA **(f)** V gene segments across groups, addressing whether GVHD is associated with biased V gene usage. Dots represent individual samples; asterisks indicate statistically significant differences. TRB, T cell receptor β; TRA, T cell receptor α; GVHD, graft versus host disease; CDR3, complementarity-determining region 3.

The analysis of CDR3 amino acid lengths across all productive reads in the samples obtained from patients with or without GVHD and HIs revealed no notable differences (**Figure 1a**, **Supplementary Figure 1a**). However, the count of clonotypes with low frequencies (less than 100) was significantly higher in patients with GVHD compared to those without GVHD (**Figures 1b, c**, **Supplementary Figure 1b**), indicating a relative enrichment of low-frequency clonotypes in GVHD patients during the early post- HSCT period. Additionally, no statistically significant differences were found in the enrichment of low-frequency clonotypes between patients with aGVHD grades ≤II and those with grades ≥III (**Supplementary Figure 2**).

To address the variations in the number of productive counts, a common limitation in TCR repertoire analyses, we performed a down-sampling procedure on the original dataset. This involved resampling the TCR data from both patients and HIs to match the lowest acceptable number of productive counts. After the normalization process, we characterized the general landscape of a “normal” immune repertoire in both patients with and without GVHD and HIs. Our results indicate that the clonotype distribution abundances exhibited no correlation with the donor’s age in patients with GVHD (**Figure 1d**, **Supplementary Figure 1c**). Distinct V and J gene usage was observed among groups, particularly for TRBV15 and TRAV2 between the GVHD and no GVHD groups. Additionally, differences in the TRAV1-1, TRAV1-2, TRAV5, TRAJ8, TRBV27, TRBV14-1, and TRBV5–6 gene segment levels were noted between the GVHD and HI groups. In addition, differences in the TRBV15 levels were also noted between the no GVHD and HI groups (**Figures 1e, f**; **Supplementary Figures 1d, e**).

### TCR repertoires exhibited increased diversity in patients with GVHD

**Figure 2:**
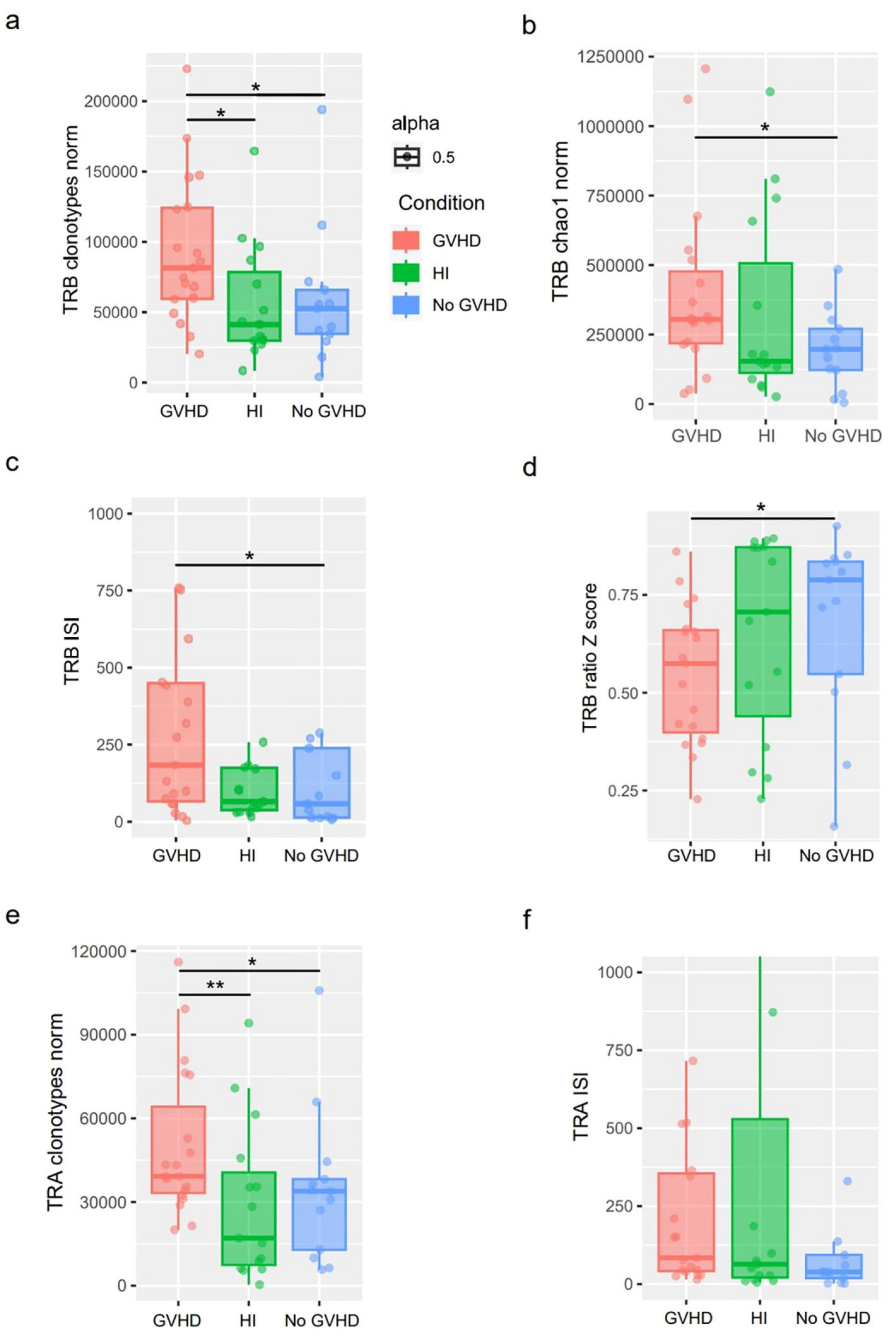
Quantitative assessment of TCR repertoire richness and diversity across clinical groups. **(a, b)** TRB repertoire richness evaluated by normalized clonotype counts **(a)** and Chao1 estimator **(b)**, addressing whether overall repertoire size differs between GVHD, no-GVHD, and healthy individuals. **(c, d)** TRB repertoire diversity assessed by the Inverse Simpson Index **(c)** and Z-score–based clonotype expansion metrics **(d)**, quantifying clonal evenness and dominance patterns across groups. **(e, f)** TRA repertoire richness **(e)** and diversity **(f)**, evaluated using normalized clonotype counts and the Inverse Simpson Index, respectively, examining whether diversity patterns observed in TRB are also present in the TRA chain. Boxplots indicate median and interquartile range; whiskers represent 1.5× IQR. Statistical comparisons were performed between groups as indicated. ISI, Inverse Simpson Index; clonotypes_norm, The number of normalized clonotypes; HI, Healthy individuals; *p<0.05, **p<0.01..

The results indicated that the numbers of TRB and TRA clonotypes in the GVHD group were significantly higher than those in the no-GVHD group and the HI group (**Figures 2a, b, e**). The clonotype frequency distribution from all samples was transformed to z-scores. Clonotypes with a z-score greater than 2 were selected. The results of the clonotype analysis indicated that the GVHD group had fewer expanded clonotypes compared to the other two groups (**Figure 2d**). A comparative analysis using the Inverse Simpson Index (ISI) showed that the TRB and TRA diversity in the GVHD group was higher than that of the no-GVHD group (**Figures 2c, f**). The GVHD and HI groups showed similar richness and diversity in the TCR repertoire, while the TCR diversity of the no GVHD group showed much lower richness and diversity. Additionally, the numbers of TRA clonotypes in the GVHD group were higher than those in the no GVHD and HI groups, while the TRA diversity exhibited a similar trend (**Figures 2e, f**). We also analyzed the distribution of top TCR clones. We found that the no GVHD group exhibited an uneven and screwed TCR clonotype distribution, while the GVHD group showed similar trends for all the properties. The top 10 intervals of the TRB counts relative to the total counts of the no-GVHD group were significantly higher than those observed in the GVHD group (**Supplementary Figure 3a**). Conversely, for the top 11 to 100, and for the 101 to 1000 TRB counts relative to total counts, the GVHD group showed an increasing trend, although not significant (**Supplementary Figure 3b, c**). Compared with the other two groups, the GVHD group had slightly higher TCR diversity in the low-frequency range (0–0.1%) but slightly lower TCR abundance at high frequencies above 0.1% (**Supplementary Figure 3d**). However, the differences were not statistically significant. Importantly, these observations were made during early immune reconstitution (day 21–100 post-HSCT) and in bulk peripheral blood T cells, and thus may reflect composite effects of immune recovery, inflammation, and immunosuppression rather than GVHD-specific clonotypes alone.

### Shared clonal motifs across patients with GVHD

**Figure 3:**
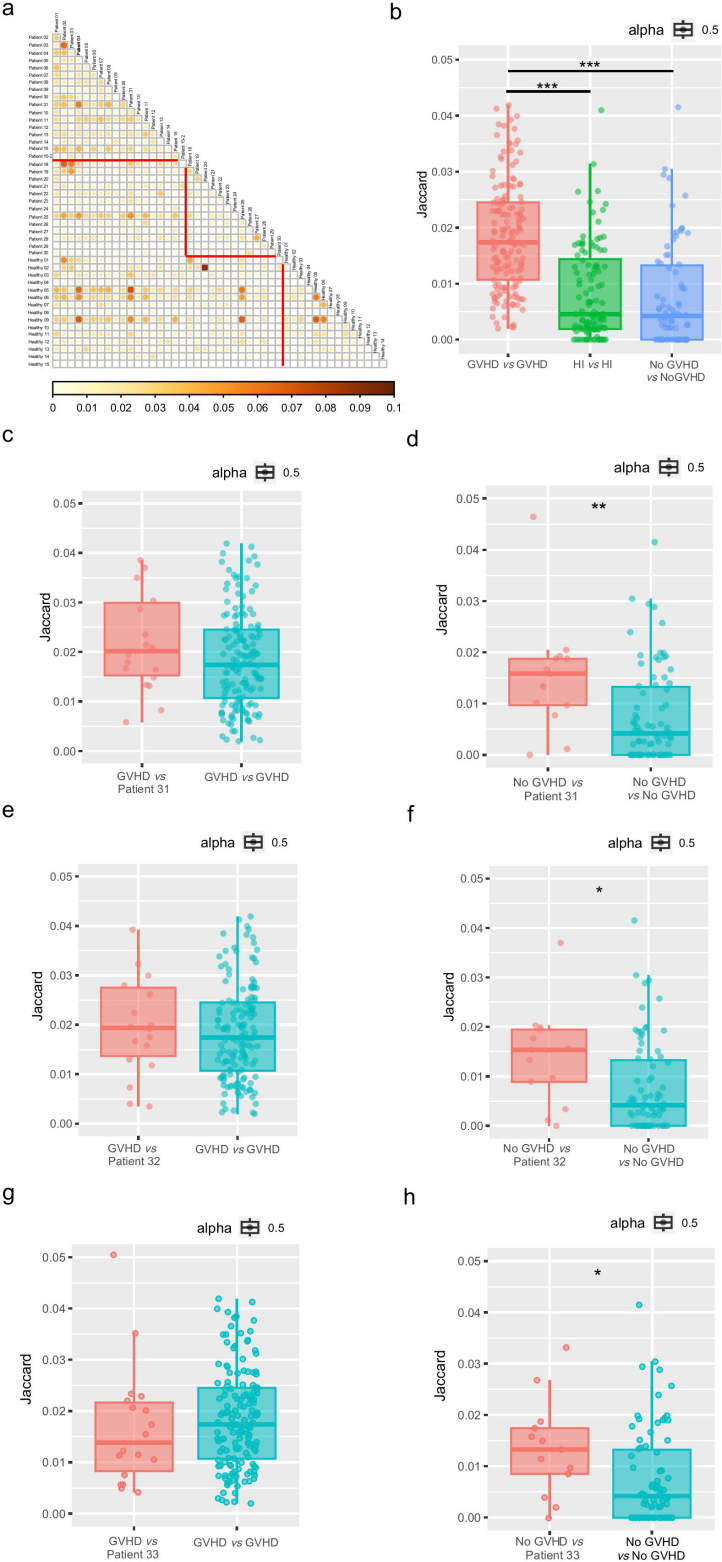
Comparison of sequence similarities for patients with GVHD or without GVHD and healthy individuals. **(a)** Pairwise comparison of the GLIPH groups. The triangular plot depicts the pairwise comparison of the GLIPH groups across patients according to the Jaccard similarity index. Higher values indicate more similarity. The triangle outlined in red on the top indicates similarities between patients with GVHD; The triangle outlined in red at the bottom right indicates similarities between patients without GVHD. The magnitude of the value is represented by the size of the circle and the intensity of its fill color simultaneously; larger circles and darker shades indicate higher similarities. **(b)** Jitter Boxplot illustrating the pairwise comparison between GLIPH groups: GVHD, HI and No GVHD. The points in the boxplots indicate the Jaccard similarity indices between two samples. The Jitter Boxplot showing the Jaccard similarity indices between GVHD patients and subject Patient 31 **(c)**, Patient 32 **(e)** and Patient 33 **(g)** while the right column shows the indices between GVHD patients themselves. The Jitter Boxplot showing the Jaccard similarity indices between no GVHD patients and subject Patient 31 **(d)**, Patient 32 **(f)** and Patient 33 **(h)** while the right column shows the indices between no GVHD patients themselves. GLIPH, Grouping of Lymphocyte Interactions by Paratope Hotspots algorithm. *p<0.05; **p<0.01; ***p<0.005.

Given the TCR clonal diversity among individuals, we explored whether there are common characteristics in the patients’ TCR in the same groups and across different groups, to identify any similar reactivity patterns. To evaluate this, we made use of the published Grouping of Lymphocyte Interactions by Paratope Hotspots algorithm version 2 (GLIPH2) to identify amino acid patterns in different TCRs that are likely to share antigen specificity to group the lymphocyte interactions according to the paratope hotspots (13–15). This approach revealed motif groups shared across patients. To assess the extent of sharing of the GLIPH groups across patients, we quantified overlap across all pairs of different groups via the Jaccard index (**Figure 3a**), a normalized measure of overlap that considers the presence versus absence of features. As shown in the results, the highest indices were observed in different samples within the GVHD group (**Figure 3b**).

To test whether the TCR repertoires have a predictive role in the occurrence of GVHD, 3 representative patients (Patient 31, Patient 32, Patient 33) who showed no clinical manifestations of GVHD (stage 0) after HSCT at the time of sampling but subsequently developed GVHD (stage 1) were selected. By comparing their TCR profiles before GVHD onset with those of the GVHD group and the non-GVHD group, we found that their TCRs were more similar to the GVHD group (**Figures 3c–h**). These findings suggest that TCR sequence profiles may have a predictive role in the occurrence of GVHD.

### Diversity of TCR repertoires decreased with amelioration of GVHD

**Figure 4:**
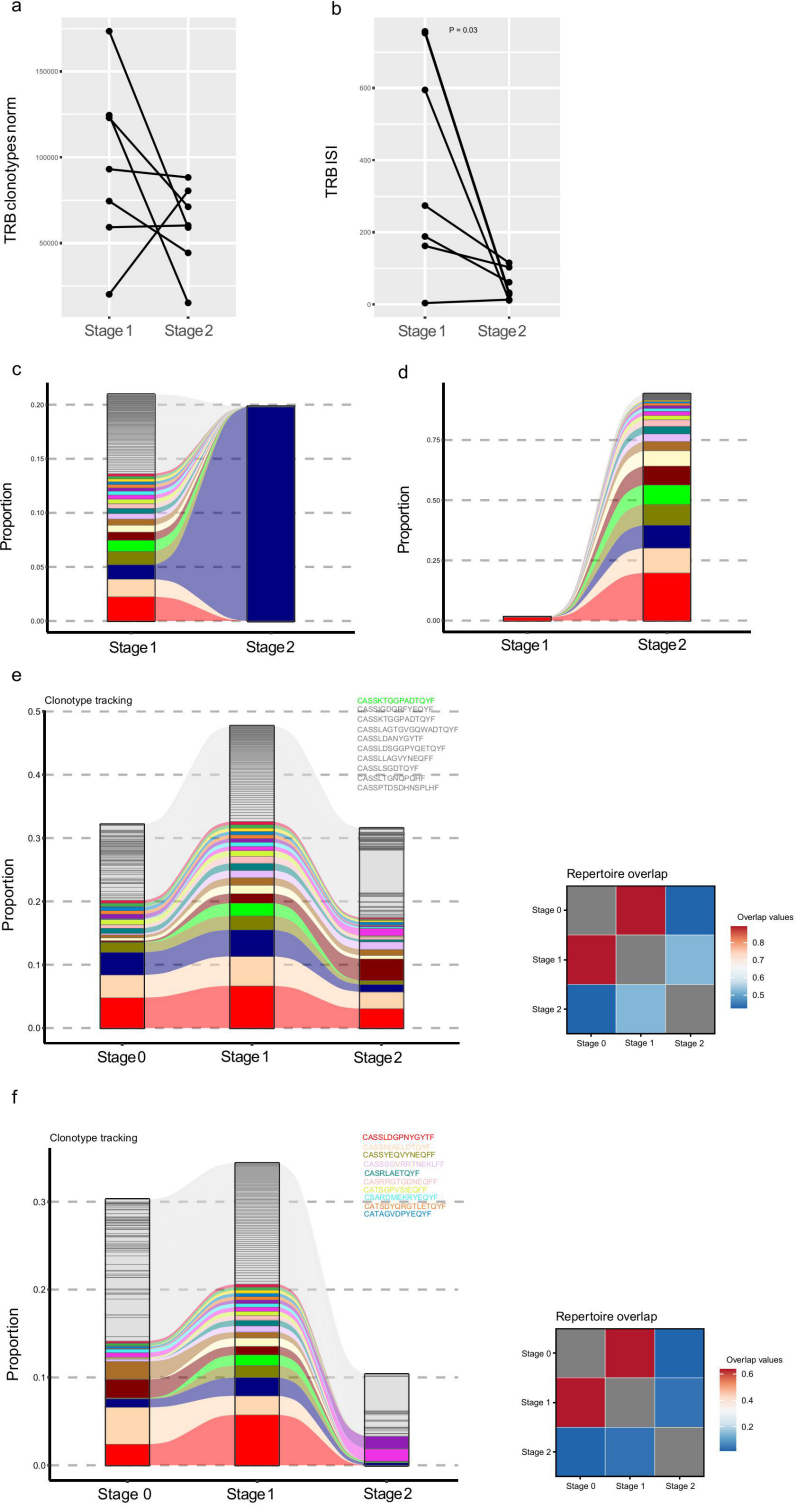
Dynamic changes in the TRB repertoire during GVHD progression (stage 1) and amelioration (stage 2). **(a)** The TRB clonotype richness of most GVHD patients in this study decreased after disease remission. **(b)** The TRB diversity decreased after disease remission for six of the observed patients. Clonotype tracking of the top 100 most abundant clones for a representative patient (Patient 04) with GVHD (stage 1) and after the amelioration of GVHD (stage 2). Top 100 clonotypes of stage 1 **(c)** or stage 2 **(d)** were used to perform clonal tracking. The colors indicate the top 20 clones. Clonotype tracking of the top 100 most abundant clones for patient 31 **(e)** and patient 32 **(f)** (left panel) who did not show GVHD at stage 0 and showed GVHD at stage 1. Then GVHD was ameliorated at stage 2. CDR marked in the upper right corner indicates clones that were among the top 20 when GVHD occurred but disappeared when GVHD resolved. The square plot depicts the pairwise comparison of the TRB clonotypes similarity across different stages of GVHD according to the Morisita index (right panel).

To further investigate the relationship between the TCR repertoire and the progression of GVHD, we compared samples from the same patient in the GVHD group at different stages: during GVHD (stage 1), and after GVHD resolution (stage 2). The results indicated that in the majority of patients (except for one patient) the number of TRB and TRA clonotypes declined and the ISI declined as the GVHD resolved, suggesting that the TCR clonotypes become less homogeneous (**Figures 4a, b**, **Supplementary Figure 4**). We further sorted the top 100 clonotypes by their abundance at different stages of GVHD occurrence and compared their presence and percentage through clonotype tracking. It was observed that more expanded clonotypes emerged after GVHD resolution, which is consistent with the previously mentioned decrease in TCR repertoire diversity (**Figures 4c, d**).

Interestingly, in two patients initially without GVHD who subsequently developed GVHD (as mentioned before, Patient 31, Patient 32), we observed that the dominant clones at the onset of GVHD diminished or disappeared as the GVHD resolved. After comparing the TCR clonotype differences across different GVHD stages we observed that the TCR clonotypes during the pre-GVHD (stage 0) and acute GVHD (stage 1) stages exhibited high similarity, whereas the TCR clonotypes during the resolution phase of GVHD (stage 2) show lower similarity to those of the previous stages (**Figures 4e, f**). This suggests that these clones are associated with GVHD and may aid in predicting its occurrence, allowing for the earlier identification of subclinical GVHD patients before symptoms manifest.

### Analysis of organ and antigen-specific TCRs in GVHD recipients

**Figure 5:**
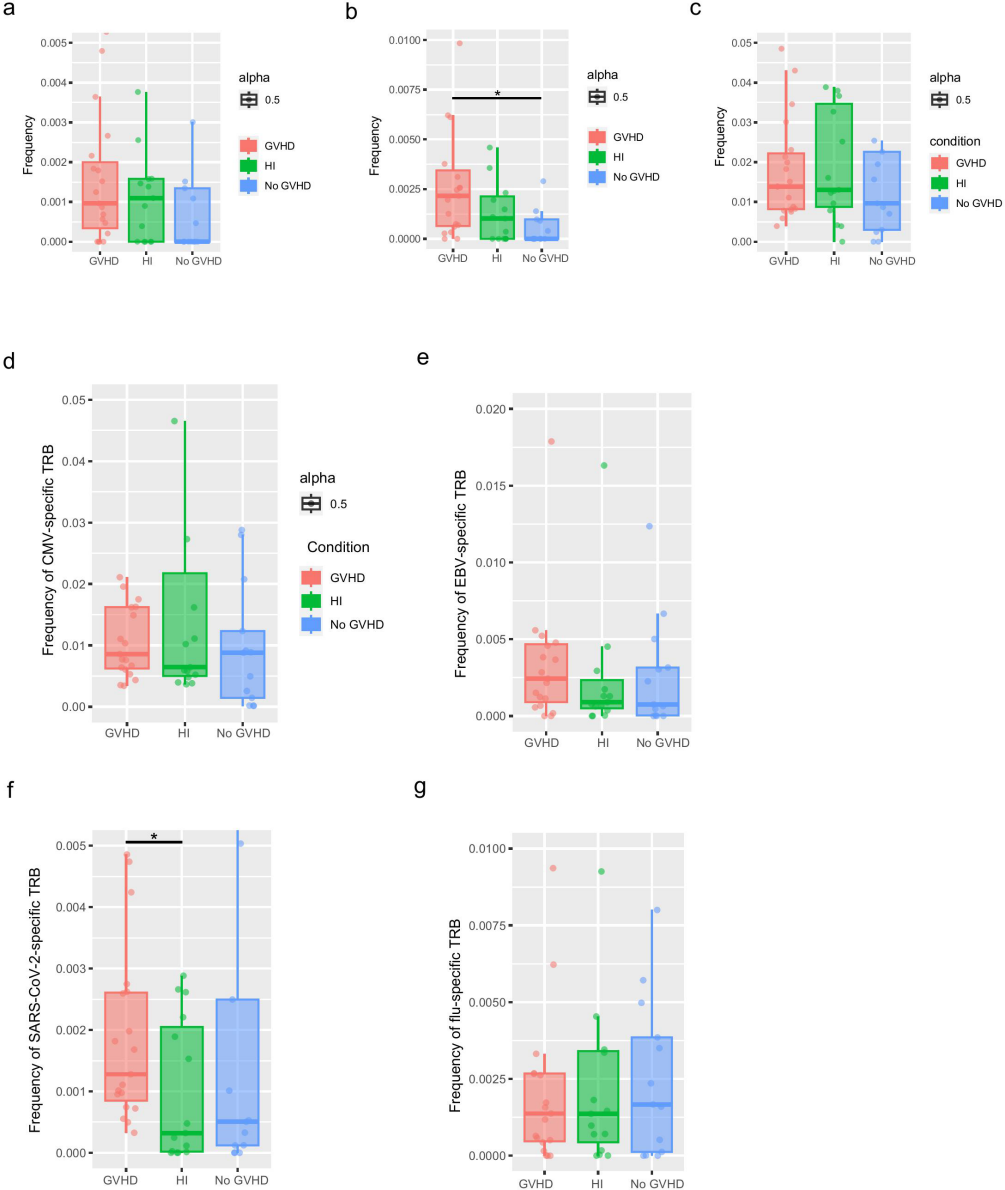
Sequence alignment with TCR sequences from published literature of GVHD identified shared TCR clones. Frequency of shared TCR clonotypes found in the blood **(a)**, boom marrow **(b)** and intestines **(c)** of GVHD patients from previous studies. Frequency of shared clonotypes in CMV–specific TRB sequences **(d)**, EBV–specific TCRβ sequences **(e)**, SARS-CoV-2–specific TCRβ sequences **(f)** and influenza–specific TCRβ sequences **(g)**. *p<0.05.

DeWolf et al. analyzed tissue samples from 7 GVHD patients, including blood, bone marrow, and intestines, to reveal specific expression patterns of TCRs in GVHD patients. Their study found specific TCR repertoire overlap across tissues and among different GVHD patients, with the same TCR sequences being expressed at higher frequencies across different patients (16). We first identified high-frequency TCR clones (CDR3s with frequency >0.01%) from the peripheral blood of our patient cohort (both GVHD and non-GVHD), then compared these clones with publicly available TCR repertoires derived from intestine, blood, and bone marrow samples of GVHD patients obtained via rapid autopsy from DeWolf’s study. For each tissue type, we computed the intersection of CDR3s with our high-frequency clones and summed their counts to evaluate the degree of clonal sharing. The validation studies in bone marrow samples showed that these same TCRs were expressed at higher proportions in GVHD patients compared to non-GVHD patients (**Figure 5b**). A similar trend was also observed in the blood (**Figure 5a**) and intestine (**Figure 5c**) samples. This result suggests that specific TCR clones may play a crucial role in the pathogenesis of GVHD. By comparing the TCR expression profiles of GVHD patients with those of non-GVHD patients, we were able to identify TCR sequences that are highly enriched in GVHD, which may be closely related to the pathological process of GVHD.

Compared to the no GVHD group, the patients in the GVHD group had higher levels of cytomegalovirus (CMV)-specific TCR, Epstein-Barr Virus (EBV)-specific TCR, and Severe Acute Respiratory Syndrome Coronavirus 2 (SARS-CoV-2)-specific TCR, while the trend for influenza-specific TCR was reversed. However, the differences were not statistically significant between the two groups (**Figures 5d–g**). This phenomenon may be associated with the use of immunosuppressants in GVHD patients, which makes these patients more prone to developing infections.

## Discussion

aGVHD arises when donor-derived T-cells identify host antigens presented on HLA molecules at the cell surface as foreign, subsequently activating and proliferating to become effector T-cells that inflict damage on host cells (17). Hence, a comprehensive understanding of T-cell reconstitution and its function in transplant recipients is crucial in revealing the molecular mechanism associated with the development of aGVHD. Nowadays, the development of highly specific diagnostic tools for predicting aGVHD remains an unresolved challenge. In the present study, we applied next-generation sequencing technology to comprehensively analyze the TCR sequences at multiple time points after HSCT in patients with or without aGVHD for hematological malignancies and compared the changes in TCR repertoire following amelioration of GVHD.

Our data revealed that the T-cells expanded clonally in both the GVHD and non-GVHD patients. The clonotypes abundance was not affected by the donor’s age in patients with GVHD. Previous studies have identified advanced donor age as a key factor impeding immune recovery, likely due to a reduced prevalence of thymic T-cell progenitors in the graft (18, 19). However, it is important to note that the difference in stem cell sources and time points of observation may also contribute to variations in the clonality abundance. The majority of GVHD cases in our study received haploidentical transplantation and their disease progression was evaluated within 100 days post-HSCT. However, in the study of Buhler et al., most patients underwent HLA-matched HSCT, with follow-up conducted one-year post-transplantation (19). Consistent with the report by Heijst et al. (8), we also found a higher TCR repertoire diversity in patients with GVHD. This consistency not only strengthens the reliability of our findings but also corroborates a phase-dependent model of GVHD pathogenesis. According to this model, early aGVHD involves broad, polyclonal activation of peripheral T cells, whereas the later or refractory stages are marked by more focused, oligoclonal expansions of tissue-resident T-cell populations (20). Importantly, the increased peripheral diversity observed in early aGVHD is not inconsistent with the oligoclonality seen in later-stage tissues; rather, it reflects the distinct immunological phases that characterize the dynamic progression of GVHD.

Studies have shown that the repertoire diversity became remarkably low in patients after the transplant (21, 22), which might be related to the conditioning regimen and/or post-transplant immune suppression. It is reasonable to assume that the higher TCR diversity in the GVHD patients was caused by donor-imprinted specificities and the expansion of certain T-cell populations might have key roles in the development of GVHD. Indeed, we observed the expansion of specific clonotypes in patients who initially showed no clinical manifestations of aGVHD but subsequently developed aGVHD. These expansions diminished or disappeared following the resolution of aGVHD.

The GLIPH2 algorithm is a proven method for analyzing TCR clustering (13). Compared to healthy individuals and recipients without GVHD, aGVHD patients exhibited higher TCR similarity in peripheral blood T cells, while the TCR similarity between healthy individuals and transplant recipients without GVHD was significantly lower than in aGVHD patients. In this study, we have demonstrated that patients exhibit higher similarity in their TCR profiles to those within the GVHD group before the onset of clinical symptoms. This suggests that the TCR repertoire may have the potential to predict the occurrence of GVHD in high-risk populations, thus enabling physicians to take prompt preventive measures.

Our findings suggest that the application of TCR detection in allo-HSCT patients depends on the establishment of a TCR sample library for the transplant population. Building TCR libraries for aGVHD, sustained remission, and post-transplant relapse, as well as potentially for different ethnicities and tissue types, could be established. Based on this database, we could make predictions for different outcomes following allo-HSCT. For T cells expanded in the peripheral blood of post-transplant patients, TCR sequencing and clustering analysis could be performed. If the expanded TCRs are highly similar to those in the aGVHD library, it indicates an increased risk of aGVHD for the patient. Alternatively, when combined with the timing and clinical symptoms of aGVHD, it could aid in the diagnosis of aGVHD. Conversely, if the expanded TCRs are highly similar to those in the sustained remission library, the patient has a lower risk of aGVHD or a diagnosis of aGVHD cannot be confirmed. Moreover, patients with parameters indicating minimal residual disease (MRD) and expanded TCRs that closely resemble those in the post-transplant relapse library may be at a higher risk of early disease relapse. However, larger sample sizes are needed to validate the reliability of our findings.

In accordance with the theoretical framework that CDR3 clonotypes serve as indicators of antigen-specific immune responses, we conducted an extensive analysis of TCR repertoires by leveraging databases of clonotypes with established specificities. While most clonotypes recognized unidentified epitopes, we successfully identified a range of sequences associated with aGVHD and linked to specific pathogens such as CMV, EBV, and SARS-CoV-2. Consistent with prior studies of CMV (19, 23), these antigen-driven clonal expansions followed structured pathways: larger clonotype sizes in later phases were linked to pathogen and tumor surveillance, whereas initial stages, particularly in GVHD, featured the proliferation of potentially autoreactive specificities. These dynamics are crucial for understanding progression toward either immunotolerance or alloreactive complications.

Extending from these antigen-specific findings to the broader repertoire level, it is important to note that the clinical interpretation of TCR diversity after HSCT is complex. Although increased diversity is often viewed as beneficial for antimicrobial and antitumor immunity, our analysis suggests its relationship with clinical protection is likely non-linear and phase-specific. In the early post-transplant period, elevated diversity may reflect a state of generalized immune dysregulation rather than the establishment of effective, targeted immune surveillance. Therefore, while our study delineates specific reactive clonotypes and contextualizes diversity patterns, future investigations with larger cohorts and extended follow-up are necessary to systematically determine how the dynamics of TCR diversity correlate with definitive clinical outcomes such as infection and disease relapse.

This study has some limitations that have to be acknowledged. The T cell count in some samples was occasionally too low, leading to fewer detectable TCR clones. As a result, due to the limited cell numbers, all repertoire analyses in this study were performed on bulk peripheral blood T cells without separation into CD4/CD8 or naïve/memory subsets. Differences in subset composition—such as relative proportions of naïve, effector, or memory T cells—can substantially influence apparent repertoire diversity, particularly during early post-transplant immune reconstitution. Therefore, the observed diversity differences should be interpreted in the context of global T-cell reconstitution dynamics rather than as changes within a specific T-cell subset. Additionally, the study was limited by a small sample size and a limited number of follow-up time points, focusing solely on aGVHD. cGVHD typically occurs later and may require more extended follow-up periods.

## Conclusion

Our study demonstrates that TCR repertoires exhibit increased diversity in patients with GVHD. This diversity decreases as GVHD improves. Our findings suggest that TCR repertoires may have predictive potential for the occurrence of GVHD, as common characteristics were observed between patients in the GVHD group.

## Materials and methods

### Study design and patients

Human specimens were studied as part of a prospective observational study whose objective was to characterize the T cell repertoire in blood samples from allo-HSCT patients with or without a history of GVHD, and from 15 healthy individuals. The study design did not include blinding or randomization. The clinical features of 15 patients with GVHD and 13 patients without GVHD were compared as shown in **Table 1**. None of the patients exhibited active CMV or EBV infections at the time of blood sample collection. Pre-transplant conditioning varied according to patient age, diagnosis, remission status, extent of prior therapies, and co-morbidities; and the dosage and intensities of the myeloablative regimens (24). GVHD prophylaxis was administered using a calcineurin inhibitor combined with short-term methotrexate, with anti-thymocyte globulin added based on protocol or physician discretion. Posttransplant granulocyte colony-stimulating factor was used in all patients. Acute and late acute GVHD were diagnosed clinically with histological confirmation when possible. Staging of GVHD was graded according to standard criteria (25, 26). All patients underwent allo-HSCT at the Department of Hematology of The First Affiliated Hospital of Soochow University between 2022 and 2023. All normal samples were obtained from healthy volunteer donors. All subjects provided Institutional Review Board-approved informed consent for collection of blood samples.

### Sample preparation and immune repertoire sequencing

Blood samples were collected at the time of aGVHD onset within 21 to 100 days following transplantation. In addition, blood samples were also collected after GVHD remission. The mononuclear cells were isolated by density centrifugation from the blood samples using a conventional protocol (8). The recovered cells were lysed in RLT buffer (QIAGEN), homogenized using QIA shredder columns (QIAGEN), and stored at −80 °C. The immune repertoire amplification and sequencing procedure described by van et al. (8) was modified as follows. Briefly, the mRNA of the TCR and BCR were purified and reverse transcribed using 5’RACE, and the V region of the TCR and BCR were amplified using two rounds of polymerase chain reaction (PCR). The specific reverse primers complementary to the C-region of the TCRs combined with an adapter were included in the reaction. The entire volume of the cDNA (10 µl) was used as a template for the 50 µl RACE-PCR. The generated amplicons were then added to the sequencing adaptors using paired-end Illumina sequencing. The raw data extraction was performed with PyIR—a minimally-dependent high-speed wrapper for the immunoglobulin Basic Local Alignment Search Tool (IgBLAST) (v1.20.0). The default options were used for the TCR sequence alignment and assembly. Before the downstream analysis, all samples were normalized to the same number (1 million) of productive reads to avoid bias as indicated in the study by Odak et al. (20). All bioinformatics and statistical analysis were performed on a Linux workstation equipped with the CentOS 7 operating system. The complete mapped sequencing data were transferred into immunarch for advanced analyses (https://immunarch.com/). The TCR clonality and diversity indices commonly used include clonal numbers/types, TCR repertoire richness/evenness (Chao1/Inverse Simpson Index), unique CDR3 species, and CDR3 sequence frequency were extracted and calculated. All TCR indices used in this study were calculated according to previous publications (8, 18, 27). Z-score normalization refers to the process of normalizing every value in a dataset such that the mean of all of the values is 0 and the standard deviation is 1.

### Statistical analysis and reproducibility

All statistical analyses were conducted using the R software version 4.2.2. The Fisher’s exact test, two-sample Kolmogorov–Smirnov test, and Wilcoxon signed rank test were used to compare the variables of TCR repertoire between different groups. The results were illustrated using boxplots. The boxplots display the median, first quartile, and third quartile, with whiskers extending to the most extreme values within 1.5 times the interquartile range (IQR). For all statistical tests, a p-value below 0.05 was deemed statistically significant.

### GLIPH motif grouping

The GLIPH2 algorithm was applied to a dataset composed of all patient samples simultaneously (TCRs defined by the Vβ CDR3 amino acid sequences and whose counts constitute more than 0.01% of the total counts), with the following GLIPH2 parameters: simulation_depth = 1000, kmer_min_depth = 3, cdr3_length_cutoff = 8, refer_file = refererence_files/ref_CD48.txt. HLA data were not included. This approach identified 5,609 GLIPH groups, including groups defined by either local (“motif”) similarities or “global” similarities. These groups were included in subsequent analyses.

### TCR sequencing alignment

The CDR3 regions of our samples were compared to an existing database of TCR sequences with known antigen specificity. The database called VDJdb can be accessed at https://vdjdb.cdr3.net and https://github.com/antigenomics/vdjdb-db and was downloaded for the comparison mentioned here.

## Data Availability

The data presented in the study are deposited in the OMIX repository (https://ngdc.cncb.ac.cn/omix/release/OMIX015287) at the National Genomics Data Center (NGDC), China National Center for Bioinformation (CNCB), under accession number OMIX015287.

## Glossary

aGVHD: Acute Graft-versus-Host Disease
HSCT: Hematopoietic Stem Cell Transplantation
TCR: T Cell Receptor
TRA: T Cell Receptor Alpha
TRB: T Cell Receptor Beta
CDR3: Complementarity-Determining Region 3
allo-HSCT: Allogeneic Hematopoietic Stem Cell Transplantation
HI: Healthy Individuals
PCR: Polymerase Chain Reaction
IgBLAST: Immunoglobulin Basic Local Alignment Search Tool
IQR: Interquartile Range
ISI: Inverse Simpson Index
GLIPH2: Grouping of Lymphocyte Interactions by Paratope Hotspots algorithm version 2
CMV: Cytomegalovirus
EBV: Epstein-Barr Virus
SARS-CoV-2: Severe Acute Respiratory Syndrome Coronavirus 2
MRD: Minimal Residual Disease
