# Supplementary material for: Quantitative evaluation of T-cell repertoire restoration following hematopoietic stem cell transplantation in patients with and without graft versus host disease
Source: Front Immunol. 2026 Mar 3;17:1778172. doi: 10.3389/fimmu.2026.1778172 (PMC12991999; doi:10.3389/fimmu.2026.1778172)
Supplement: Supplementary file 1 [file DataSheet1.pdf]

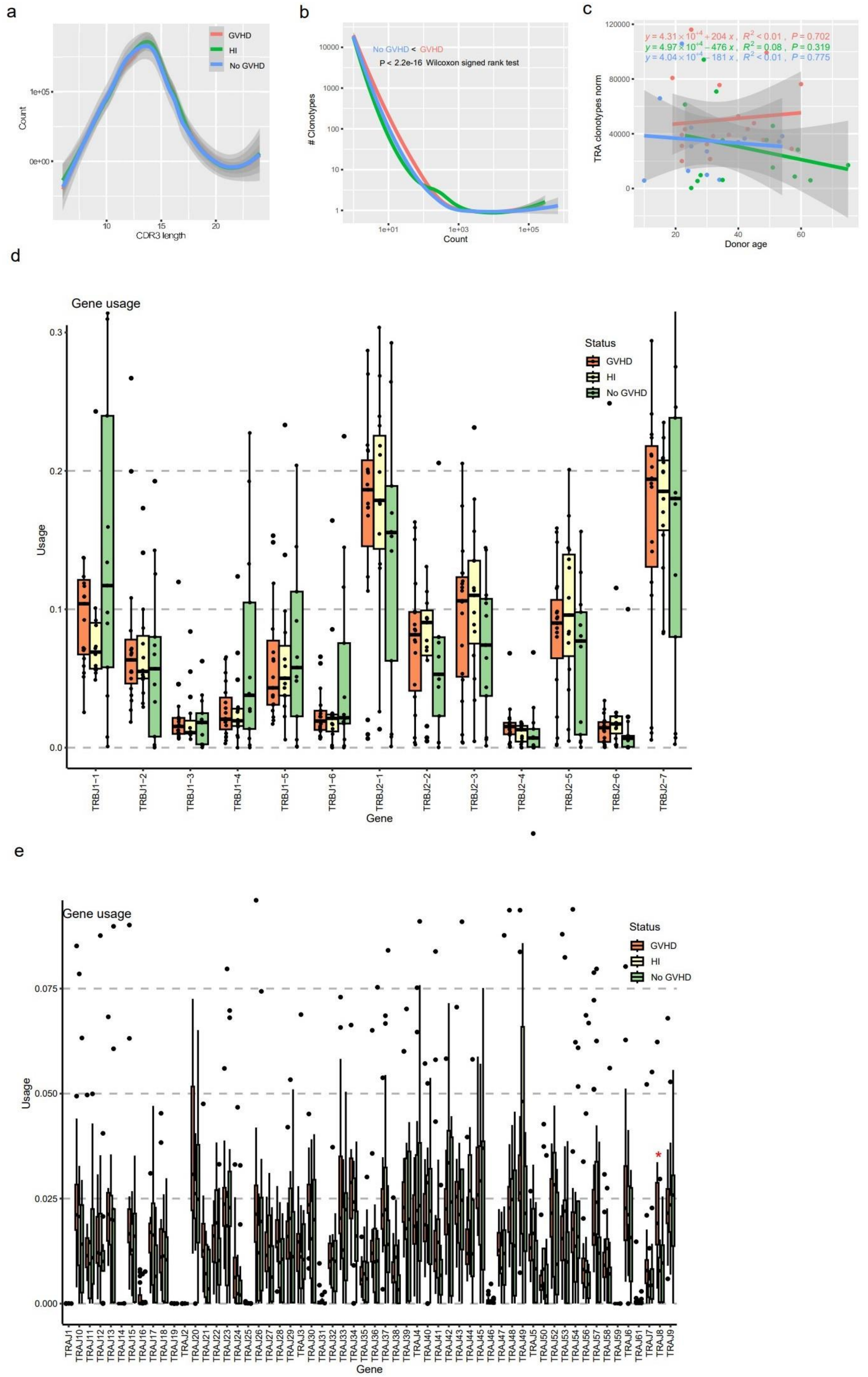

Supplementary figure 1: TRA repertoire analysis for patients with or without

GVHD and healthy donors

(a) Distribution of the CDR3 amino acid lengths in TRA for all productive reads for each sample. The 95% confidence interval for predictions from a linear model computed by the geom_loess function is shown in grey.

(b) Distribution of clonotype abundances (smooth norm) in TRA. The numbers of clonotypes with low counts (count < 100) in patients with GVHD were higher than those of patients without GVHD (one-sided Kolmogorov–Smirnov test and Wilcoxon signed rank test). The 95% confidence interval for predictions from a linear model computed by the geom_loess function is shown in grey.

(c) Pearson correlations between age and TRA clonotype richness in patients and healthy donors. The 95% confidence interval for predictions from a linear model computed by the geom_loess function is shown in grey.

Distribution of the TRB chain J gene usage (d) and TRA chain J gene usage (e) for each group. The dots indicate the outliers.

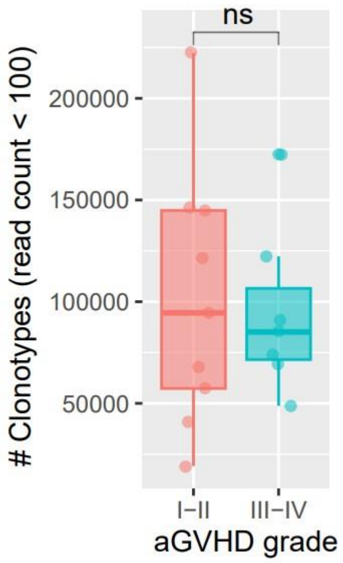

Supplementary figure 2: Comparison of low-frequency clonotypes (read count <

100) in patients with different grades of aGVHD.

No statistically significant differences were observed in numbers of low-frequency clonotypes (count <100) between patients with aGVHD grades ≤II and those with grades ≥III. ns, not significant.

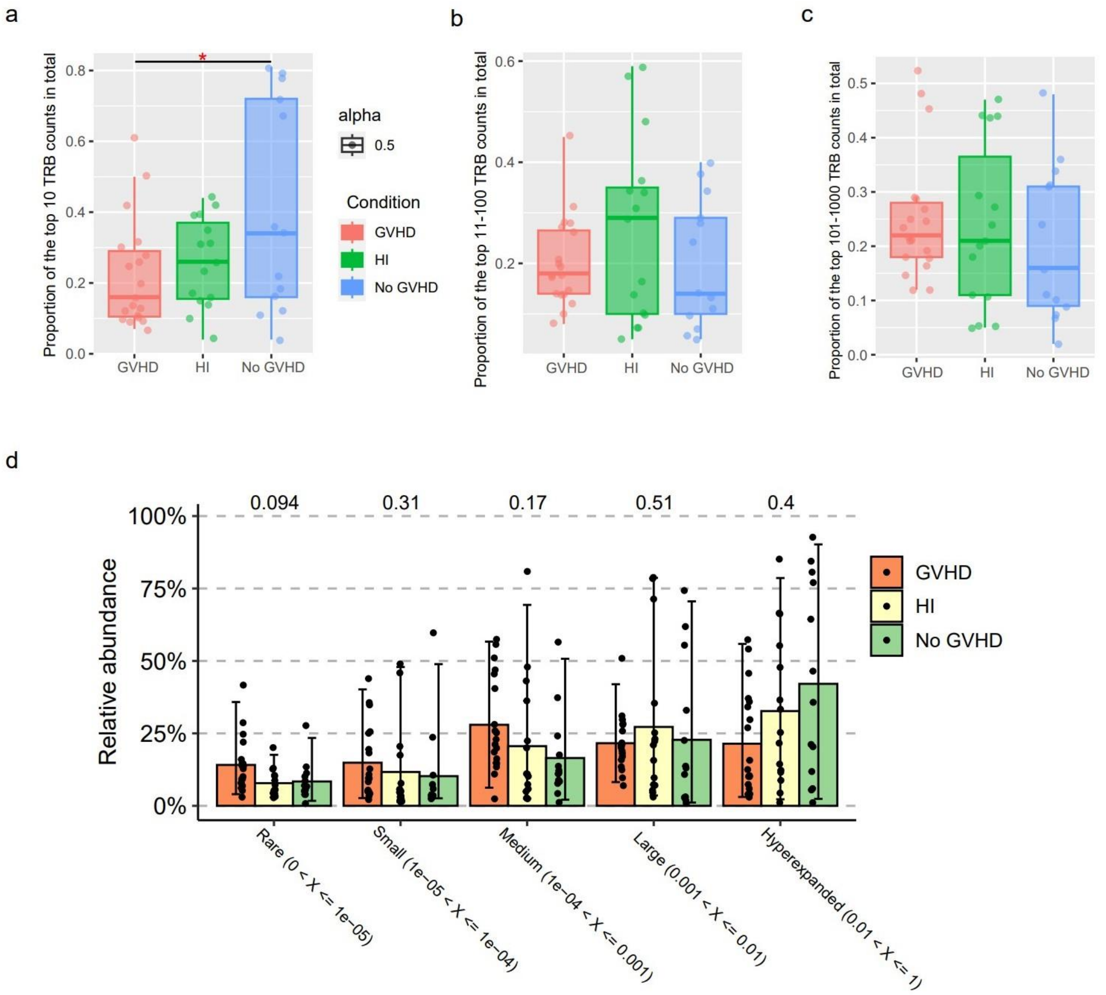

Supplementary figure 3:

Boxplots illustrating the accumulated frequency (dots) of the top expanded clonotypes for the top 10 TRB counts (a), the top 11 to 100 TRB counts (b), and the top 101 to 1000 TRB counts (c). The boxplots display the median, first quartile, and third quartile, with whiskers extending to the most extreme values within 1.5 times the interquartile range (IQR). (d) Visualization of clone proportions from the total repertoire for the rare clones (0-0.1%), small clones (0.1-1%), medium clones (1-10%), and expanded clones

(10-100%).

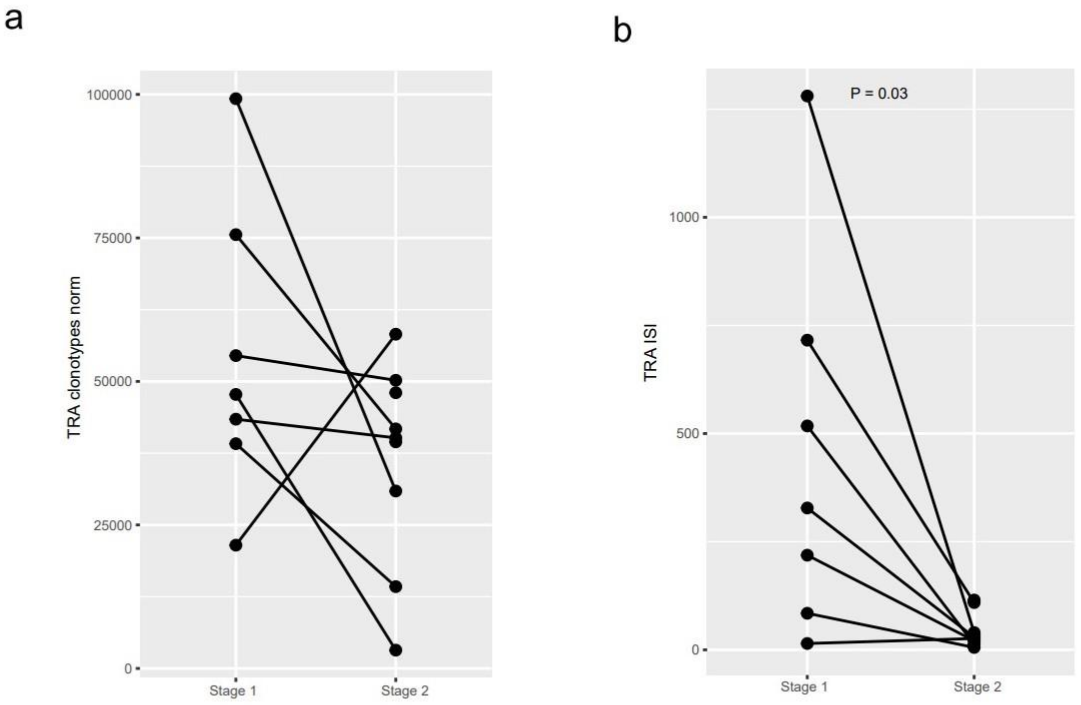

Supplementary figure 4: Dynamic changes in the TRA repertoire during GVHD progression (stage 1) and amelioration (stage 2)

(a) The TRA clonotype richness of GVHD patients in this study decreased after disease remission. (b) The TRA diversity decreased after GVHD disease remission.
